# Anti-3-Hydroxy-3-Methylglutaryl-Coenzyme A Reductase (HMGCR) Immune-Mediated Necrotizing Myopathy With Subsequent Diagnosis of Mantle Cell Lymphoma

**DOI:** 10.7759/cureus.108086

**Published:** 2026-05-01

**Authors:** Chuchu Zhao, Amy Duffield, Bahtiyar Toz

**Affiliations:** 1 Department of Medicine, NYC Health + Hospitals/Queens, Icahn School of Medicine at Mount Sinai, New York, USA; 2 Department of Pathology, Molecular and Cell-Based Medicine, Icahn School of Medicine at Mount Sinai, New York, USA

**Keywords:** anti-hmgcr myopathy, hematologic malignancy, hyperckemia, immune-mediated necrotizing myopathy, mantle cell lymphoma, proximal muscle weakness, statin-associated myopathy

## Abstract

Anti-3-hydroxy-3-methylglutaryl-coenzyme A reductase (anti-HMGCR) immune-mediated necrotizing myopathy (IMNM) is a subtype of autoimmune necrotizing myopathy that presents with progressive proximal weakness and persistent creatine kinase (CK) elevation, which may continue despite statin discontinuation. Its relationship with hematologic malignancy is not well established.

A 67-year-old woman developed markedly elevated CK levels after starting high-intensity statin. Despite statin withdrawal, CK levels remain persistently elevated, accompanied by progressive symmetrical proximal weakness. Anti-HMGCR IgG was strongly positive, while other autoimmune serologies were unrevealing. A diagnosis of statin-associated anti-HMGCR IMNM was established, and treatment with mycophenolate mofetil and intravenous immunoglobulin resulted in partial biochemical improvement.

Five weeks after initiation of immunosuppressive therapy, routine laboratory testing showed rapidly rising leukocytosis. Further evaluation revealed mantle cell lymphoma with bone marrow involvement, IGH::CCND1 rearrangement, and *TP53* deletion, consistent with high-risk disease.

In this context, continued clinical and laboratory monitoring is appropriate in inflammatory myopathy, and new hematologic abnormalities warrant further evaluation.

## Introduction

Immune-mediated necrotizing myopathy (IMNM) typically presents with severe proximal muscle weakness and markedly elevated creatine kinase (CK) and often requires immunosuppressive therapy [1]. Anti-3-hydroxy-3-methylglutaryl-coenzyme A reductase (anti-HMGCR) IMNM is commonly statin-associated and may persist despite discontinuation of the inciting agent [2]. Recent data estimate the incidence of HMGCR-associated IMNM at approximately 2.9 cases per million person-years, with higher rates among statin-exposed individuals [3]. Despite increasing recognition, the association between anti-HMGCR IMNM and malignancy remains unclear [4,5].

While malignancy screening is well established in dermatomyositis, the strength of association between anti-HMGCR IMNM and malignancy, particularly hematologic malignancy, remains less clearly defined [6,7].

We report a case of anti-HMGCR IMNM followed by high-risk mantle cell lymphoma, highlighting the importance of continued surveillance when new hematologic abnormalities emerge.

## Case presentation

A 67-year-old woman with coronary artery disease, type 2 diabetes mellitus, and hyperlipidemia was started on high-intensity rosuvastatin for cardiovascular risk reduction. Four months later, she was hospitalized with nausea and vomiting and found to have a markedly elevated creatine kinase (CK) level peaking at 9,674 U/L, consistent with non-traumatic rhabdomyolysis. On examination, she had generalized weakness, which had been present for three months since a prior rehabilitation stay, with strength documented as 4/5 in all four extremities and no documented proximal predominance. There was no rash, skin thickening, conjunctival pallor, lymphadenopathy, or hepatosplenomegaly. Rosuvastatin was discontinued. CK declined but remained above 5,000 U/L at discharge.

CK levels remained persistently elevated for over one year, and she developed gradually worsening symmetric proximal muscle weakness despite statin withdrawal. Given her cardiovascular risk, low-dose rosuvastatin was briefly reintroduced but again discontinued when CK remained elevated. However, after three weeks off therapy, there was still no improvement in either her symptoms or CK levels, and treatment was resumed.

She was referred to rheumatology for evaluation of persistent CK elevation and progressive proximal weakness. CK was 5,745 U/L, with concurrent aspartate aminotransferase (AST) and alanine aminotransferase (ALT) elevation. Antinuclear antibody (ANA), anti-double-stranded DNA, SS-A (anti-Ro) and SSB (anti-La) antibodies, antiphospholipid antibodies, and complement levels were normal. A myositis panel demonstrated weak anti-SAE-1 positivity that was negative on repeat testing. Anti-HMGCR IgG was markedly elevated at 124.9 Units (reference range 0.0-19.9 Units).

In the setting of persistent CK elevation, progressive proximal weakness, strong anti-HMGCR positivity, and lack of improvement after statin discontinuation, a diagnosis of statin-associated anti-HMGCR IMNM was made. Femur MRI and hip MRI were recommended to further evaluate for inflammatory myopathy and guide consideration of muscle biopsy; however, the patient declined MRI, and muscle biopsy was ultimately not pursued. Although histopathologic confirmation was unavailable, the clinical and serologic findings were considered sufficient to support the diagnosis. Statins were discontinued, and treatment with mycophenolate mofetil and intravenous immunoglobulin (2 g/kg every four weeks) was initiated. Within one month, CK declined to approximately 1,800 U/L with partial symptomatic improvement. Laboratory findings at key time points are summarized in Table 1.

**Table 1:** Summary of laboratory findings during the clinical course AST: Aspartate aminotransferase; ALT: Alanine aminotransferase; ANA: Antinuclear antibody

Test	Value	Reference Range	Unit
Creatine kinase (4 months after statin initiation)	9,674	20–170	U/L
Creatine kinase (after statin withdrawal)	5,745	20–170	U/L
Creatine kinase (after immunosuppressive therapy)	1,810	20–170	U/L
AST	155	10–35	U/L
ALT	161	10–40	U/L
ANA	Negative	<1:80	—
Anti–double-stranded DNA	1	<=4	IU/mL
SS-A (Anti-Ro) antibodies	<0.2	<1.0	—
SS-B (Anti-La) antibodies	<0.2	<1.0	—
Beta 2 Glycoprotein 1 IgA Ab	<2.0	<20.0	U/mL
Cardiolipin Ab IgG, IgM and IgA	Negative	<20	U/mL
C3 Complement	148	81–157	mg/dL
C4 Complement	25	13–39	mg/dL
Anti–SAE 1 antibody	26	<20	Units
Anti-SAE 1 antibody (repeat)	<20	<20	Units
Anti-HMGCR IgG	124.9	0.0–19.9	Units
White blood cell (WBC) count (initial)	17.87	4.8–10.8	×10^3^/mcL
WBC count (peak)	62.46	4.8–10.8	×10^3^/mcL
Lymphocyte % (at peak WBC)	90.1	13.0–44.0	%
Hemoglobin (at peak WBC)	8.0	12.0–16.0	g/dL
Platelet count (at peak WBC)	53	150–450	×10^3^/mcL

**Figure 1:**
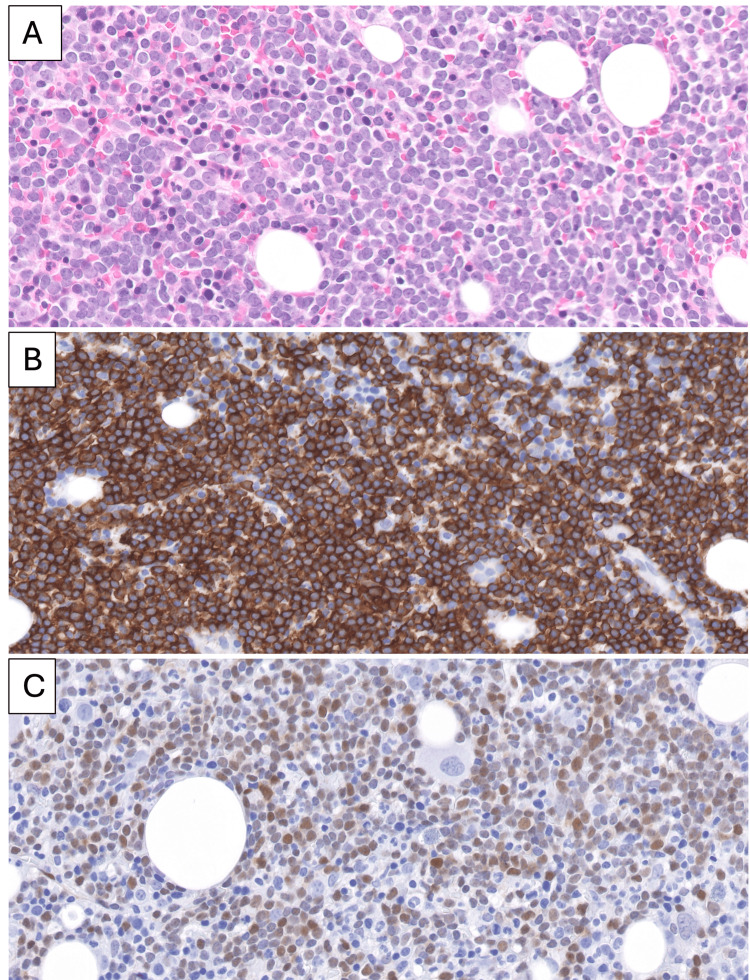
Bone marrow involvement by mantle cell lymphoma (A) Hematoxylin and eosin staining shows diffuse infiltration of the marrow by small lymphocytes with scant cytoplasm and mature chromatin. (B) Immunohistochemical stains for CD20 highlight the neoplastic lymphocytes. (C) Cyclin D1 highlights the neoplastic lymphocytes. (Magnifications ×600)

Five weeks after starting immunosuppressive therapy, routine laboratory testing revealed rapidly progressive leukocytosis (17.87 × 10^3^/mcL to 62.46 × 10^3^/mcL) with lymphocyte predominance, accompanied by worsening anemia and thrombocytopenia. She denied fever, night sweats, or unintentional weight loss, and examination showed no lymphadenopathy or hepatosplenomegaly. Peripheral blood flow cytometry demonstrated a CD5-positive, lambda-restricted clonal B-cell population. Cytogenetic testing identified a t(11;14)(q13;q32) CCND1::IGH rearrangement, and bone marrow biopsy (Figure 1) confirmed mantle cell lymphoma. Additional fluorescence in situ hybridization studies (FISH) demonstrated that the neoplastic B-cells had a deletion on the short arm of chromosome 17, which includes *TP53*, consistent with high-risk disease. The lymphoma cells were cyclin D1-positive, and the Ki-67 proliferation index was estimated at 10%-20%.

**Figure 2:**
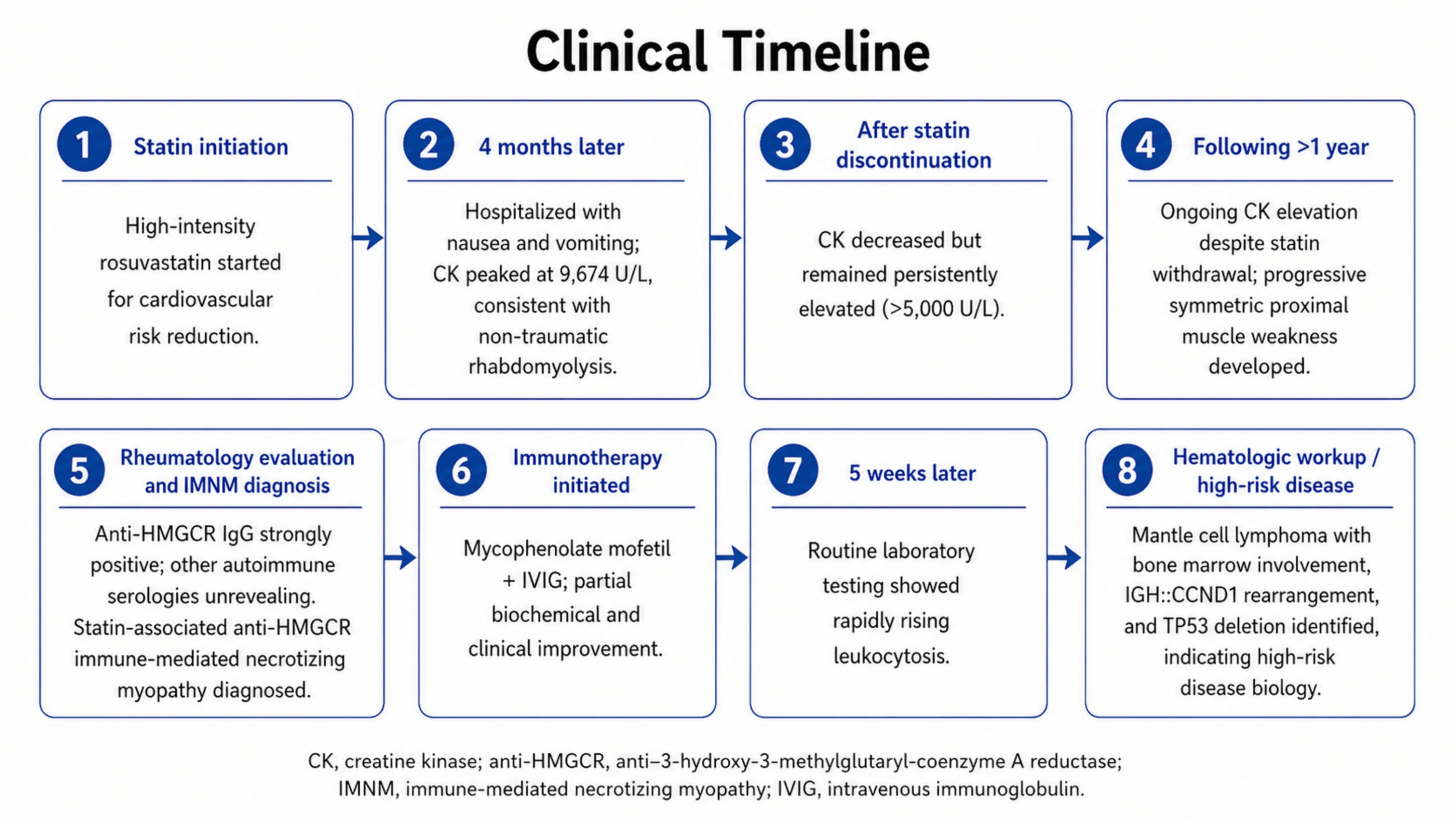
Clinical timeline of the patient’s course from statin initiation to the diagnosis of anti-HMGCR immune-mediated necrotizing myopathy and subsequent mantle cell lymphoma

She received rituximab-based immunochemotherapy (R-CHOP) initially and was later switched to obinutuzumab, zanubrutinib, and venetoclax due to high-risk disease features. Her course was complicated by significant cytopenias and infections requiring close monitoring. At the most recent follow-up, she was clinically stable but remained cytopenic and continued hematology and rheumatology follow-up with close laboratory monitoring. Formal lymphoma response assessment was pending at the time of manuscript preparation. The chronological clinical course is summarized in Figure 2.

## Discussion

Anti-HMG-CoA reductase immune-mediated necrotizing myopathy (anti-HMGCR IMNM) typically presents with progressive proximal weakness, markedly creatine kinase (CK) elevation, and anti-HMGCR autoantibodies. Although initially described in the context of statin exposure, discontinuing statin alone is often not sufficient. In many patients, weakness and CK elevation persist, and immunosuppression is often required [1]. Experimental studies indicate that statins increase HMGCR expression, and regenerating muscle fibers may continue to express the antigen after withdrawal, possibly contributing to ongoing immune activation [2]. In this patient, CK elevation and weakness continued despite repeated statin discontinuation and improved only after initiation of mycophenolate mofetil and intravenous immunoglobulin. This clinical course was more consistent with immune-mediated necrotizing myopathy than self-limited toxic statin myopathy. Although a muscle biopsy was not performed, the strongly positive anti-HMGCR antibody in the appropriate clinical setting was considered sufficient to support the diagnosis.

The relationship between anti-HMGCR IMNM and malignancy is still not clearly defined. Earlier cohort studies reported higher cancer rates in necrotizing autoimmune myopathy, particularly in anti-HMGCR-positive and seronegative patients, although no specific tumor subtype emerged [4,5]. More recent longitudinal and case-control studies have not consistently shown an increased overall cancer risk compared with matched controls [8]. Differences in patient selection, follow-up duration, and antibody stratification may account for the variation across studies. Recent international recommendations support risk-stratified malignancy screening in idiopathic inflammatory myopathies, including patients with IMNM, rather than universal intensive evaluation [7].

Data on hematologic malignancies in anti-HMGCR IMNM are limited, and most reports focus on solid tumors. Histopathologic studies have described BCL-2-positive and CCR4-positive lymphocytic infiltrates and occasional lymphocytic aggregates in muscle and skin [9,10]. These findings, however, do not demonstrate clonal proliferation or malignant transformation and are not sufficient to support a diagnosis of lymphoma.

When lymphoma and myopathy coexist, several mechanisms should be considered. Intravascular large B-cell lymphoma can involve muscle, with biopsy showing malignant lymphoid cells confined to small vessels rather than the myofiber necrosis typically seen in immune-mediated necrotizing myopathy [11-13]. In addition, inflammatory neuromuscular syndromes have been reported in association with chronic lymphoid leukemia and other lymphomas, most often presenting as dermatomyositis, polymyositis, or heterogeneous inflammatory syndromes rather than antibody-defined IMNM [14,15]. Reports of anti-HMGCR IMNM occurring with B-cell lymphoma remain limited.

Links between malignancy and autoimmunity have been described in some rheumatic diseases. In systemic sclerosis, somatic tumor mutations may trigger immune responses against shared autoantigens, suggesting a model of cancer-induced autoimmunity [16]. There are also isolated reports of IMNM associated with hematologic malignancies, raising the possibility that tumor-related immune dysregulation could contribute to loss of tolerance in some cases [17]. However, these mechanisms have not been established in anti-HMGCR IMNM, and a causal relationship remains uncertain.

In our patient, anti-HMGCR IMNM and high-risk mantle cell lymphoma were diagnosed within a short time interval. The presence of *TP53* deletion adds further complexity. *TP53* abnormalities are important adverse prognostic markers in mantle cell lymphoma and are associated with aggressive disease biology, poor response to conventional chemoimmunotherapy, and shorter progression-free and overall survival [18,19]. In this case, *TP53* deletion supported high-risk disease biology; however, its relevance to anti-HMGCR IMNM remains unclear, and a causal relationship cannot be established. Still, new or evolving hematologic abnormalities during follow-up should prompt careful evaluation rather than being attributed solely to immunosuppressive therapy. Whether this reflects coincidence or represents shared or interacting disease mechanisms remains uncertain. Close collaboration between rheumatology and hematology is important in managing such patients.

## Conclusions

Anti-HMGCR IMNM can be diagnosed in close temporal proximity to mantle cell lymphoma. A causal relationship cannot be determined. In patients with inflammatory myopathy, new or evolving hematologic abnormalities should prompt further evaluation rather than be attributed solely to therapy. Coordination between rheumatology and hematology is often required when these conditions coexist.
